# Dihydromyricetin Protects against Diabetic Cardiomyopathy in Streptozotocin-Induced Diabetic Mice

**DOI:** 10.1155/2017/3764370

**Published:** 2017-03-21

**Authors:** Bin Wu, Jie Lin, Jian Luo, Dong Han, Miaomiao Fan, Tao Guo, Ling Tao, Ming Yuan, Fu Yi

**Affiliations:** ^1^Department of Cardiology, Xijing Hospital, Fourth Military Medical University, Xi'an, Shaanxi 710032, China; ^2^Department of Internal Medicine (VIP), The First Affiliated Hospital, Xinjiang Medical University, Urumqi, Xinjiang 830000, China

## Abstract

Diabetic cardiomyopathy (DCM) is an important cause of heart failure in diabetic patients. The present study sought to explore the potential effects of dihydromyricetin (DHM) on DCM and its possible mechanism. A diabetic model was induced by intraperitoneal injection of streptozotocin (STZ) in C57BL/6J mice. Two weeks after the STZ injection, mice were randomly allocated into the following 4 groups for treatment: the control group (CON), the control treated with DHM group (CON + DHM), the diabetes group (DM), and the diabetes treated with DHM group (DM + DHM). DHM was dissolved in distilled water and administered daily by gavage. For 14 weeks, the CON + DHM group and DM + DHM group were given a dose of 100 mg/kg/day DHM (Sigma-Aldrich), while the CON and DM groups were intragastrically given equivalent volumes of distilled water. Assessments and comparisons were made among the groups based on cardiac function and structural changes, inflammation factors, markers of oxidative stress, mitochondria function, apoptosis, and autophagy. The DHM treatment normalized body weight, preserved cardiac function, attenuated oxidative stress (MDA, SOD, and GSH-Px), reduced the levels of inflammation factors (IL-6, TNF-*α*), alleviated pathological changes, improved mitochondrial function (ATP content, CS activity, and complex Ι/ΙΙ/ΙΙΙ/ΙV/V activities), inhibited cardiac apoptosis, and restored autophagy in diabetic mice. DHM may have a great therapeutic potential in the treatment of DCM.

## 1. Introduction

Diabetes is an increasingly serious global health burden. An estimated 382 million people had diabetes in 2013, and this number is expected to rise to 592 million by 2035 [1]. Diabetes increases the risk of developing heart failure in the absence of coronary heart disease and hypertension, resulting in a structural and functional impairment termed diabetic cardiomyopathy (DCM) [2, 3]. DCM leads to early-onset diastolic and late-onset systolic dysfunction in type 1 and type 2 diabetes. There are many important contributors to diabetic cardiomyopathy onset and progression, such as left ventricular hypertrophy, metabolic abnormalities, extracellular matrix changes, small vessel disease, oxidative stress, apoptosis, and autophagy [4]. However, no effective strategy alleviates the progression of DCM, and the mechanisms of DCM have been poorly elucidated.

*Ampelopsis grossedentata* has been widely used as a traditional Chinese herb in the south region of China. Dihydromyricetin (DHM) is the most abundant and bioactive flavonoid component of* Ampelopsis grossedentata*. Over hundreds of years, DHM has been proven to have numerous pharmacological activities, including antioxidant, anti-inflammation, antihypertension, anticancer, hepatoprotection against alcohol intoxication, and antifatigue activities [5]. DHM can prevent adriamycin-induced cardiotoxicity by protecting myocardial cells from apoptosis and attenuate angiotensin II-induced rat cardiomyocyte hypertrophy in an NO-dependent manner [6, 7]. Moreover, DHM can mimic the effects of exercise-induced irisin secretion, which may be of great benefit to patients who suffer from metabolic diseases [8]. Additionally, DHM can improve skeletal muscle insulin resistance by inducing autophagy and facilitating glucose and lipid metabolism in patients with nonalcoholic fatty liver disease [9, 10]. More importantly, DHM exerts antioxidant effects, which may contribute to its cardiovascular protective effects. DHM protects human umbilical vein endothelial cells from hydrogen peroxide-induced oxidative stress damage by regulating mitochondrial pathways [11]. Although accumulating evidence indicates that DHM exerts cardiovascular protective effects and is good for metabolic diseases, the possible beneficial effects of DHM on DCM have not yet been fully elucidated.

In the present study, we sought to demonstrate the potential effects of DHM on cardiac dysfunction and possible mechanisms in streptozotocin- (STZ-) induced diabetic mice.

## 2. Materials and Methods

### 2.1. Experimental Animals and Diabetes Model

Eight-week-old male C57BL/6 mice (weight, 25–30 g) were purchased from the Laboratory Animal Center of the Fourth Military Medical University. All animal protocols were approved by the Fourth Military Medical University Ethic Committee on Animal Care, and all experiments were performed in adherence with the National Institutes of Health Guidelines on the Use of Laboratory Animals. Experimental diabetic mice were induced by intraperitoneal (i.p.) injection of streptozotocin (STZ) (50 mg/kg, STZ was dissolved in 0.1 mM citrate buffer, pH 4.5) for 5 days. Animals with glucose levels no less than 16.6 mmol/L were classified as diabetic (DM).

### 2.2. Experimental Protocol

Two weeks after the STZ injection, nondiabetic and diabetic mice were randomly selected and allocated into the following 4 groups (30 mice in each group): the control group (CON), the control treated with DHM group (CON + DHM), the diabetes group (DM), and the diabetes treated with DHM group (DM + DHM). DHM was dissolved in distilled water and administered daily by gavage. The CON + DHM and DM + DHM groups were given a dose of 100 mg/kg/day DHM (Sigma-Aldrich) for 14 weeks, while the CON and DM groups were intragastrically given equivalent volumes of distilled water. We chose the rational dose of DHM (100 mg/kg/d) according to the literature [9, 12]. After 14 weeks, M-mode echocardiography was performed, and the mice were sacrificed.

### 2.3. Echocardiography

Echocardiography was conducted by M-mode echocardiography using an echocardiography system with a 15-MHz linear transducer (VisualSonics Vevo 2100, Toronto, Canada). Two-dimensional guided M-mode measurements of the LV internal diameter were obtained from the short-axis view at the level of the papillary muscles over at least three beats and were averaged. Computer algorithms were used to calculate LV end-diastolic dimension (LVEDD), LV end-systolic dimension (LVESD), left ventricular ejection fraction (LVEF), and left ventricular fraction shortening (LVEF).

### 2.4. Determination of Tissue Interleukin-6 and Necrosis Factor-Alpha Activity

The heart tissue samples were homogenized (1 : 10, w/v) in Tris-HCl buffered solution (10 mM, pH 7.4) and then centrifuged at 10000 ×g for 20 min at 4°C. The resultant supernatant was used for the cytokine assessment. The concentrations of interleukin-6 (IL-6) and tumor necrosis factor-alpha (TNF-*α*) were assessed using ELISA kits (Uscn Life Science Inc., Wuhan, China) according to the manufacturer's instructions. Values are expressed as pg/mg of total protein.

### 2.5. Estimation of Superoxide Dismutase (SOD), Malondialdehyde (MDA), and Glutathione Peroxidase (GSH-Px) in Heart Tissue

The heart tissue samples were weighed and then homogenized (1 : 10, w/v) in phosphate buffer (50 mM, pH 7.4). The levels of SOD, GSH-Px, and MDA were assessed by colorimetric analysis using a spectrophotometer and the appropriate detection kits (Nanjing Jiancheng Bioengineering Research Institute, China).

### 2.6. Tissue Collection and Histology

Mouse hearts were excised at the end of the experiments. Left ventricles were fixed in 4% buffered paraformaldehyde, paraffin-embedded, and sectioned at 5 *μ*m. At least 10 random sections were selected from each group, stained with hematoxylin-eosin (H&E), visualized by light microscopy, and photographed. The degree of fibrosis was detected with Masson trichrome to stain the heart sections for collagen. Quantification of the blue area, representing collagen, was performed with Photoshop software (Adobe, Seattle, WA, USA).

### 2.7. Electron Microscopy

Heart tissues were removed from the animal with ophthalmic scissors and quickly rinsed in PBS at a low temperature. A sample removed from the left ventricular myocardium was cut into 1 mm cubes. After fixation, soaking, stepwise alcohol dehydration, displacement, embedding, polymerization, sectioning, staining, and observation with an electron microscope (JEM-2000EX TEM, Tokyo, Japan), images were taken. Random sections were photographed and analyzed by two technicians who were blinded to the treatments.

### 2.8. Determination of Mitochondrial Transmembrane Potential (ΔΨ*m*)

Tetrachloro-tetraethyl benzimidazol carbocyanine iodide (JC-1) was used to determine the changes in ΔΨ*m*. JC-1 was added to cardiomyocytes that were cultured on confocal dishes; the cells were then incubated for 30 min in the dark and washed twice with PBS. JC-1-labeled cells were observed under an Olympus FV1000 laser confocal microscope. The JC-1 aggregates that displayed red fluorescence represent high ΔΨ*m*, whereas green fluorescence represents low ΔΨ*m*.

### 2.9. Mitochondria Isolation

Mitochondria were isolated from hearts as previously described [13]. The mouse hearts were excised and rinsed with ice-cold medium A (120 mM NaCl, 2 mM MgCl_2_, 20 mM HEPES, 1 mM EGTA, and 5 g/l bovine serum albumin; pH 7.4) to remove any residual blood. Cardiac tissue was minced in ice-cold medium A and homogenized. Then, the homogenate was centrifuged (600 ×g, 10 min, 4°C), and the subsequent supernatant was centrifuged again (17,000 ×g, 10 min, 4°C). The pellet containing the mitochondria was resuspended in medium A and then centrifuged (7000 ×g, 10 min, 4°C), and the resulting pellet was resuspended in medium B (2 mM HEPES, 300 mM sucrose, 0.1 mM EGTA; pH 7.4) and recentrifuged (3500 ×g, 10 min, 4°C). The final pellet containing the heart mitochondria was suspended in a small volume of medium B and stored at −80°C until use. All work was performed on wet ice at 0°C.

### 2.10. Citrate Synthase, Chain Complex Activities, and ATP Content

Prior to chain complex activity measurements, all sample homogenates were subjected to 3 freeze-thaw cycles that would disrupt the mitochondrial membranes and allow substrates access to the active sites of the enzymes. Citrate synthase (CS) and electron transport chain complex activities were measured using a CS activity assay kit (Sigma-Aldrich, USA). The ATP content of the myocardium was measured using an adenosine triphosphate bioluminescent assay kit (Beyotime, Shanghai, China).

### 2.11. Western Blot Analysis

After rinsing with PBS, heart tissues and cells were lysed on ice for 30 min in lysis buffer containing a protease inhibitor cocktail (Roche, MD, USA). After centrifugation at 12000 ×g for 15 min, the supernatant was separated and stored at 80°C until use. The protein concentration was determined using a BCA protein assay kit (Applygen, Beijing, China). Protein samples were subjected to PAGE and transferred to PVDF membranes. The membranes were blocked, incubated with the above-indicated primary antibodies, and then incubated with HPR-conjugated secondary antibodies. Immunoreactive bands were scanned and detected by a chemiluminescence system (Amersham Bioscience, Buchinghamshire, UK).

### 2.12. Reagents and Antibodies

The following antibodies were used: P62, Beclin1 (Abcam, Cambridge, MA, UK), cleaved caspase-3, cleaved caspase-9, Bax, Bcl-2 (Sigma, St. Louis, MO, USA), LC3A/B, Atg7, p-AMPK (Thr172), AMPK, p-ULK1 (Ser757), ULK1 (Cell Signaling, Danvers, MA, USA), *β*-actin (Santa Cruz, CA, USA), and horseradish peroxidase-conjugated secondary antibodies (anti-mouse/rabbit IgG) (Cell Signaling).

### 2.13. Neonatal Mouse Ventricular Cardiomyocyte Culture and Transfection

Primary cultures of cardiomyocytes were harvested from the ventricle of neonatal (1-day-old) C57BL/6 mice. The neonatal mice were sterilized with 75% ethanol, and the hearts were removed rapidly and rinsed in cold PBS to flush out the remaining blood. The myocardial specimens were then cut into pieces and digested with collagenase type 2 (Sigma-Aldrich) until the tissue blocks had disappeared. The suspension was collected and mixed with complete medium to stop the digestion. Then, the mixture was centrifuged at 800 ×g for 5 min, and the supernatant was removed. The cardiomyocyte-rich fraction was resuspended in complete medium containing 20% fetal bovine serum (Gibco, California, USA). Differential adhesion was used to effectively separate the cardiomyocytes from fibroblasts. The dissociated cells were plated in a culture flask at 37°C for 1 h to ensure that the fibroblasts could adhere to the bottom of the culture flask, and the cardiomyocytes were replated on confocal dishes. The cardiomyocytes were cultured in low glucose (5.5 mM, LG) or high glucose (33 mM, HG) DMEM supplemented with 20% fetal bovine serum and 1% penicillin-streptomycin at 37°C in the presence of 95% O_2_ and 5% CO_2_. After 48 h, the cells were treated with DHM (1 *μ*M) in the absence or presence of high glucose for 24 h. Cultured cardiomyocytes were treated with 50 nM bafilomycin A1 (Sigma-Aldrich) for 2 h to evaluate the autophagy flux.

### 2.14. Cell Transfection

Adenovirus harboring GFP-LC3 (GFP-LC3) was purchased from GeneChem Technology Ltd. (Shanghai, China). The cardiomyocytes were incubated with the transfection mixture for 48 h and supplemented with fresh medium for an additional 24 h. The indicated adenovirus was transfected according to the manufacturer's protocol.

### 2.15. Detection of GFP-LC3

Actin filaments were stained with rhodamine-conjugated phalloidin, which was purchased from Cytoskeleton. GFP-LC3 fluorescence was observed under an Olympus FV1000 laser confocal microscope. The number of GFP-LC3 puncta was counted in five independent visual fields.

### 2.16. Detection of Aggresomes and P62

The ProteoStat Aggresome Detection Kit was used to detect aggregated proteins within the aggresome and aggresome-like structures. The procedures were conducted according to the manufacturer's protocol. The aggresome and aggresome-like structures were stained red with the ProteoStat aggresome red fluorescent molecular dye. P62 was probed with a primary anti-P62 antibody and a secondary antibody labeled with Alexa Fluor 647 dye (Life Technologies). Actin filaments were stained with FITC-conjugated phalloidin (Cytoskeleton). The colocalization of aggresomes and P62 was detected with an Olympus FV1000 laser confocal microscope.

### 2.17. Immunofluorescence Analysis

Cells were fixed with 4% formaldehyde in DMEM for 15 min at 37°C. The fixed cells were then permeabilized with 0.2% Triton X-100 (Sigma, T8787) for 15 min on ice, blocked, incubated with primary antibodies overnight at 4°C, and then incubated with secondary antibodies at room temperature for 1 h. Cell images were captured with a laser-scanning confocal microscope (Olympus FV1000).

### 2.18. Determination of Cardiomyocyte Apoptosis

Terminal deoxyribonucleotidyl transferase-mediated dUTP-biotin nick end labeling (TUNEL) was used to determine cardiomyocyte apoptosis. TUNEL staining was performed with fluorescein-dUTP (In Situ Cell Death Detection Kit; Roche Diagnostics) to identify apoptotic cell nuclei, and 4,6-diamidino-2-phenylindole (DAPI) (Sigma) stained all cell nuclei. Additional staining was performed using a monoclonal antibody against Troponin I (cTnI, Santa Cruz) to identify the myocardium. The apoptotic index was calculated as the percentage of the TUNEL-positive cells divided by the total number of DAPI-positive cells within the same area from five randomly selected fields for each treatment.

### 2.19. Statistical Analysis

Data from at least three independent experiments are expressed as the means ± SDs. Statistical comparisons among different groups were evaluated using one-way ANOVA followed by the Student-Newman-Keuls text. The level of significance was set at *P* < 0.05. SPSS software package version 14.0 (SPSS, Chicago, IL, USA) was used for data analysis.

## 3. Results

### 3.1. Treatment with DHM Normalizes Body Weight in STZ-Induced Diabetic Mice

The blood glucose was more than 16.6 mmol/L in the diabetic group and was significant compared with control group before DHM treatment. During the experimental period of 14 weeks, the blood glucose remained at this level in the diabetic group. In parallel with the increased blood glucose, general characteristics such as reduced body weight, increased food consumption, and water intake appeared in the diabetic group compared with the control and DHM-treated control groups. DHM supplementation enhanced body weight but did not affect the blood glucose, food consumption, or water intake of diabetic mice (Table 1).

### 3.2. Treatment with DHM Improves Cardiac Function in STZ-Induced Diabetic Mice

**Figure 1:**
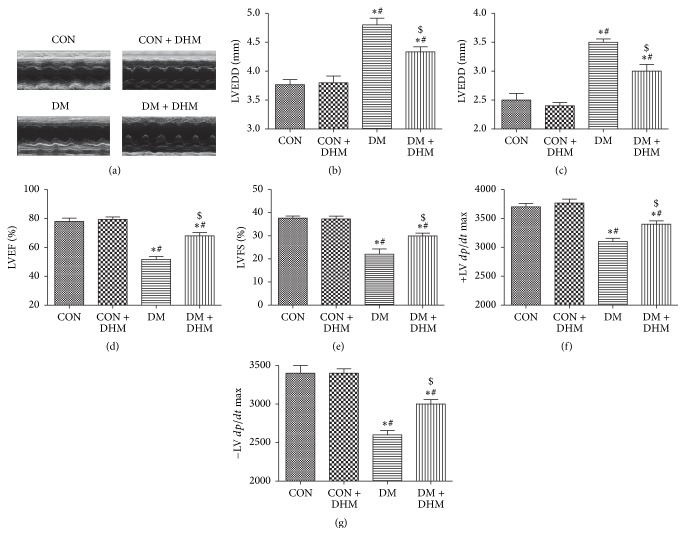
Effects of DHM on cardiac function in STZ-induced diabetic mice. (a) Representative echocardiographic images are shown. (b) LV end-diastolic diameter (LVEDD). (c) LV end-systolic diameter (LVESD). (d) LV ejection fraction (LVEF). (e) LV fraction shortening (LVFS). ((f) and (g)) First derivative of the LV pressure (±LV *dp*/*dt* max). The columns and error bars represent the means and SD (*n* = 6). ^*∗*^*P* < 0.05 versus CON; ^#^*P* < 0.05 versus CON + DHM; ^$^*P* < 0.05 versus DM.

To investigate the effect of DHM treatment on the cardiac function of diabetic mice, echocardiography was used to measure cardiac function parameters (Figure 1(a)). After 14 weeks of diabetes, LVEDD and LVESD significantly increased in the diabetic group compared with control group. DHM treatment did not affect the LVEDD and LVESD of the control group; however, it suppressed increases in the diabetic group, and this effect was significant compared with that of the untreated diabetic group (Figures 1(b) and 1(c)). LVEF, LVFS, and ±LV *dp*/*dt* were lower in the diabetic group than in the control and DHM-treated control groups. Treatment with DHM increased LVEF, LVFS, and ±LV *dp*/*dt* in the diabetic group compared with the untreated diabetic group (Figures 1(d), 1(e), 1(f), and 1(g)).

### 3.3. Treatment with DHM Attenuates Oxidative Stress and Inflammation in STZ-Induced Diabetic Mice

**Figure 2:**
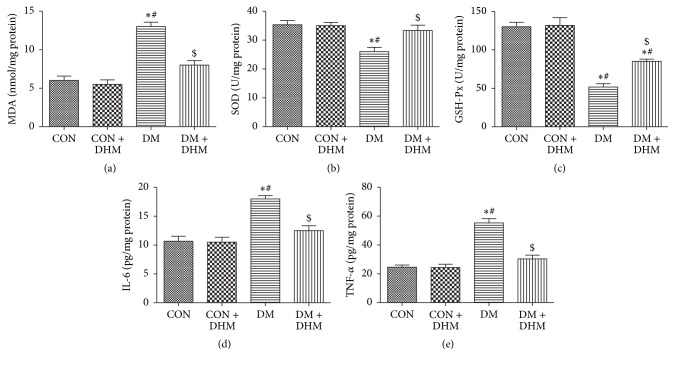
Effects of DHM on inflammation and oxidative stress in STZ-induced diabetic mice. (a) DHM decreased the MDA content in diabetic heart tissue. ((b) and (c)) DHM enhanced the activities of SOD and GSH-Px in diabetic heart issue. ((d) and (e)) DHM decreased levels of IL-6 and TNF-*α* in diabetic heart tissue. The columns and error bars represent the means and SD (*n* = 6). ^*∗*^*P* < 0.05 versus CON; ^#^*P* < 0.05 versus CON + DHM; ^$^*P* < 0.05 versus DM.

The MDA level in the myocardium was higher in the diabetic group than in the control and DHM-treated control groups, and DHM treatment decreased the MDA level in the diabetic group (Figure 2(a)). The activities of SOD and GSH-Px, which are myocardial antioxidative enzymes, were decreased in the diabetic group compared with the control and DHM-treated control groups and increased by DHM treatment (Figures 2(b) and 2(c)).

The levels of inflammatory factors IL-6 and TNF-*α* were also increased in the diabetic myocardium compared with that of the control and DHM-treated control groups. In contrast, lower levels of IL-6 and TNF-*α* were detected in the DHM-treated diabetic group (Figures 2(d) and 2(e)).

### 3.4. Treatment with DHM Alleviates Pathological Changes in STZ-Induced Diabetic Mice

**Figure 3:**
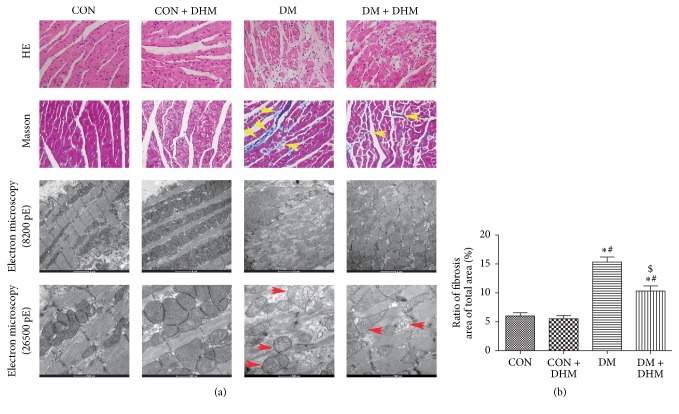
Effects of DHM on pathological changes in STZ-induced diabetic mice. (a) Representative images of myocardial tissue sections stained with hematoxylin and eosin ((a) first row, magnification = 400x); representative images of myocardial tissue sections stained with Masson's trichrome ((a) second row, magnification = 400x, yellow arrows indicate collagen deposition); representative transmission electron micrographs of left ventricular specimens ((a) third and fourth rows, magnification = 8200x and 26500x, red arrows indicate swollen mitochondria). (b) Quantification of fibrosis. The columns and error bars represent the means and SD (*n* = 6). ^*∗*^*P* < 0.05 versus CON; ^#^*P* < 0.05 versus CON + DHM; ^$^*P* < 0.05 versus DM.

H&E staining, Masson staining, and transmission electron microscopy were used to evaluate the myocardial pathological changes in STZ-induced diabetic mice (Figure 3(a)). H&E staining showed that the myocardial fibers were arranged regularly, with distinct intercellular borders in the control and DHM-treated control groups, but the arrangement of the cardiac fibers was disrupted, and the intercellular borders were obscure in the diabetic group (Figure 3(a) first row). Moreover, Masson staining showed that there was fibrosis in the diabetic group, with a disorganized collagen network structure and collagen deposition (Figures 3(a) second row and 3(b)). Transmission electron microscopy showed normal mitochondria packed beside the fibers and typical, symmetric myofibrils composed of Z lines with sarcomeres in the control and DHM-treated control mice. In contrast, swollen mitochondria and the loss of myofibrils due to disrupted sarcomere units were evident in diabetic mice (Figure 3(a) third and fourth rows). Compared with the diabetic group, DHM treatment ameliorated the pathological abnormalities mentioned above in the diabetic mice.

### 3.5. Treatment with DHM Improves Mitochondrial Function in STZ-Induced Diabetic Mice

**Figure 4:**
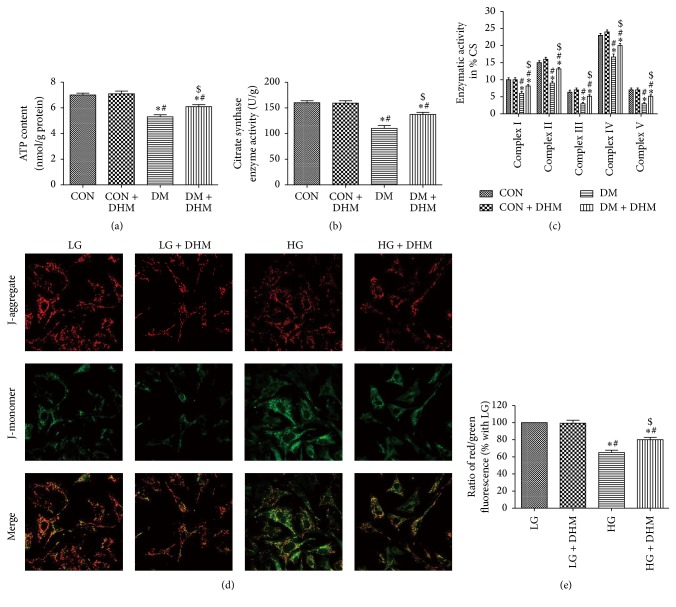
Effects of DHM on mitochondrial function in STZ-induced diabetic mice. (a)–(c) ATP content, citrate synthase (CS), and complex Ι/ΙΙ/ΙΙΙ/ΙV/V activities in isolated mitochondria from mice. The columns and error bars represent the means and SD (*n* = 6). ^*∗*^*P* < 0.05 versus CON; ^#^*P* < 0.05 versus CON + DHM; ^$^*P* < 0.05 versus DM. (d) Representative images of JC-1 staining in cardiomyocytes. (e) The ratio of aggregated and monomeric JC-1. The columns and error bars represent the means and SD. ^*∗*^*P* < 0.05 versus LG; ^#^*P* < 0.05 versus LG + DHM; ^$^*P* < 0.05 versus HG.

Mitochondrial dysfunction is associated with cardiac remodeling in diabetic mice. The ATP content, CS activity, and complex Ι/ΙΙ/ΙΙΙ/ΙV/V activities in the isolated mitochondria were significantly decreased in the diabetic group compared with the control and DHM-treated control groups. The levels of ATP content, CS activity, and complex Ι/ΙΙ/ΙΙΙ/ΙV/V activities were enhanced in the DHM-treated diabetic group compared with the diabetic group (Figures 4(a), 4(b), and 4(c)). JC-1 fluorescence in cardiomyocytes showed enhanced mitochondrial potential in the DHM-treated high glucose group compared with the high glucose group (Figures 4(d) and 4(e)).

### 3.6. Treatment with DHM Inhibits Cardiac Apoptosis in STZ-Induced Diabetic Mice

**Figure 5:**
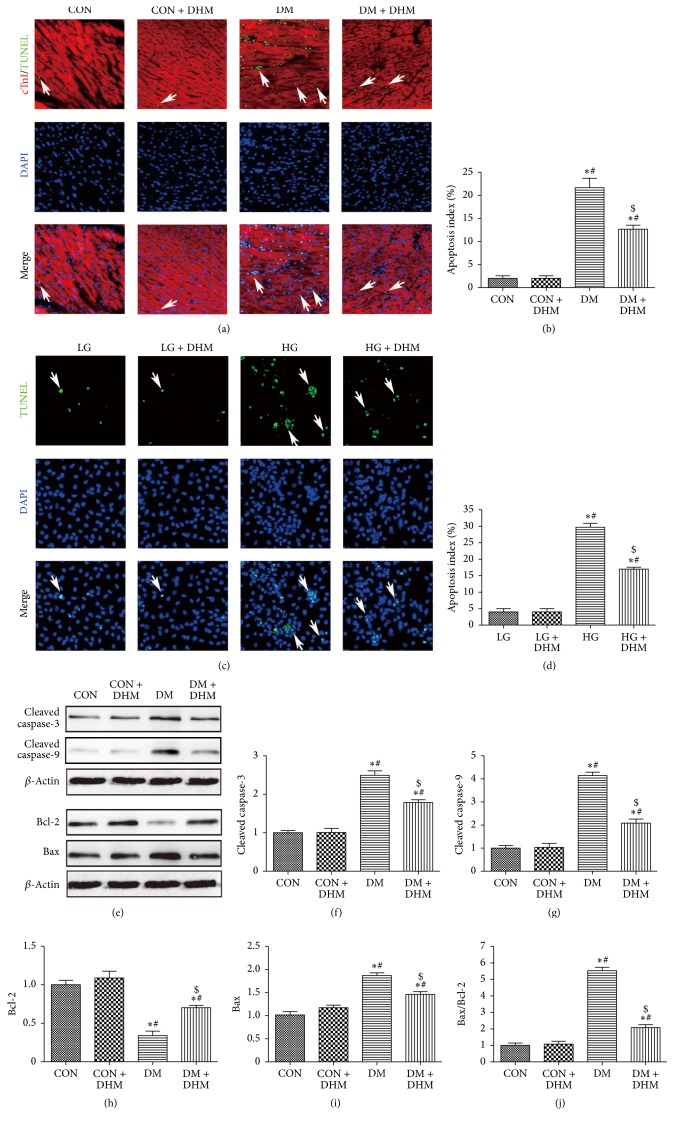
Effects of DHM on cardiac apoptosis in STZ-induced diabetic mice. (a)-(b) Representative immunofluorescent images of staining with TUNEL (green), DAPI (blue), and Troponin T antibody (red) and quantification of TUNEL-positive cells. White arrows indicate apoptotic nuclei. The columns and error bars represent the means and SD (*n* = 6). ^*∗*^*P* < 0.05 versus CON; ^#^*P* < 0.05 versus CON + DHM; ^$^*P* < 0.05 versus DM. (c)-(d) Representative images of TUNEL-stained primary neonatal cardiomyocytes and quantitative analysis of the apoptotic index. White arrows indicate apoptotic nuclei. ^*∗*^*P* < 0.05 versus LG; ^#^*P* < 0.05 versus LG + DHM; ^$^*P* < 0.05 versus HG. The columns and error bars represent the means and SD. (e)–(j) Quantitative analysis of protein expression levels in the myocardium, with representative gel blots of caspase-3, cleaved caspase-3, caspase-9, cleaved caspase-9, Bcl-2, Bax, and *β*-actin. The columns and error bars represent the means and SD (*n* = 6). ^*∗*^*P* < 0.05 versus CON; ^#^*P* < 0.05 versus CON + DHM; ^$^*P* < 0.05 versus DM.

The TUNEL assay was performed to evaluate effects of DHM on cardiac apoptosis (Figures 5(a) and 5(c)). Significantly more TUNEL-positive cardiomyocytes were detected in the diabetic group compared with the control and DHM-treated control groups. DHM treatment decreased the percentage of TUNEL-positive cardiomyocytes in diabetic mice (Figure 5(b)). Similar results were observed in in vitro studies, which showed that DHM treatment inhibited high glucose-induced cardiomyocyte apoptosis (Figure 5(d)). Western blot was used to assess caspase enzyme activity (Figure 5(e)). The levels of cleaved caspase-3 and cleaved caspase-9 were significantly higher in the diabetic group than in the control group with or without DHM treatment. DHM treatment decreased the levels of cleaved caspase-3 and cleaved caspase-9 in the myocardium of diabetic mice (Figures 5(f) and 5(g)). We also investigated the effects of DHM on the expression of the apoptosis-related Bcl-2 family by Western blot (Figure 5(e)). A higher Bax/Bcl-2 ratio, resulting from upregulated Bax protein and downregulated Bcl-2 protein, was found compared with the control group with or without DHM treatment. DHM treatment increased the Bcl-2 level and decreased the Bax level and Bax/Bcl-2 ratio of diabetic mice (Figures 5(h), 5(i), and 5(j)).

### 3.7. Treatment with DHM Restores Cardiac Autophagy in STZ-Induced Diabetic Mice

**Figure 6:**
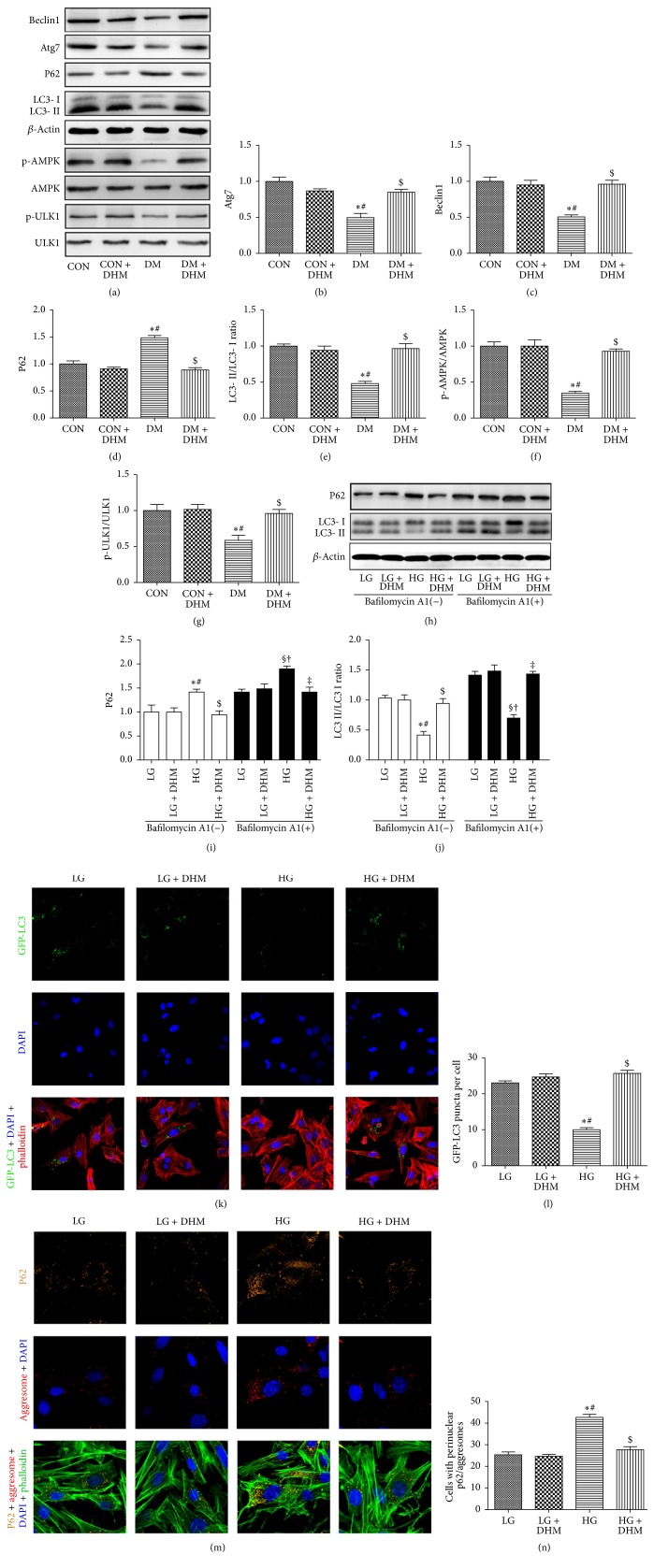
Effects of DHM on cardiac autophagy in STZ-induced diabetic mice. (a)–(g) Representative immunoblots for Beclin1, Atg7, P62, LC3 II, p-AMPK, AMPK, p-ULK1, ULK1, and *β*-actin in myocardial tissues from the respective groups and densitometric quantification. The columns and error bars represent the means and SD (*n* = 6). ^*∗*^*P* < 0.05 versus CON; ^#^*P* < 0.05 versus CON + DHM; ^$^*P* < 0.05 versus DM. (h)–(j) Representative blots and analysis of P62 and LC3 in the absence or presence of bafilomycin A1. (k) DHM increased the number of GFP-LC3 puncta in high glucose-cultured neonatal cardiomyocytes. (l) Quantitative analysis of the number of GFP-LC3 puncta. (m) DHM decreased the accumulation of P62 (orange) and aggresomes (red) in high glucose-cultured neonatal cardiomyocytes. (n) Quantitative analysis of the mean numbers of autophagosomes and autolysosomes. The columns and error bars represent the means and SD. ^*∗*^*P* < 0.05 versus LG; ^#^*P* < 0.05 versus LG + DHM; ^$^*P* < 0.05 versus HG; ^§^*P* < 0.05 versus LG + bafilomycin A1; ^†^*P* < 0.05 versus LG + DHM + bafilomycin A1; ^‡^*P* < 0.05 versus HG + bafilomycin A1.

Western blot revealed that the LC3 ΙΙ/LC3 Ι ratio and the expression of Beclin1 and Atg7 were reduced and P62 increased in the hearts of diabetic mice compared with the hearts of control mice with or without DHM treatment. DHM treatment restored the LC3 ΙΙ/LC3 Ι ratio and the expression of Beclin1 and Atg7 and decreased the P62 level of diabetic mice (Figures 6(a), 6(b), 6(c), 6(d), and 6(e)). Bafilomycin A1, a lysosomal inhibitor, was used to evaluate autophagic flux. Consistently, the enhanced autophagic flux exerted by DHM was demonstrated by an increased LC3 ΙΙ/LC3 Ι ratio and decreased P62 expression in the presence of bafilomycin A1 (Figures 6(h), 6(i), and 6(j)). In cardiomyocytes transfected with GFP-LC3, the number of green puncta found in high glucose incubation was less than in low glucose incubation. DHM treatment increased the number of green puncta in high glucose incubation (Figures 6(k) and 6(l)). Consistently, the cardiomyocytes cultured under high glucose conditions showed more accumulation of aggresomes and P62 than cardiomyocytes cultured under low glucose conditions. DHM treatment significantly reduced the accumulation of aggresomes and P62 in cardiomyocytes under high glucose culture conditions (Figures 6(m) and 6(n)).

Western blot was used to investigate AMPK (AMP-activated protein kinase) and phosphorylation of its downstream signaling target ULK1 (the mammalian homolog of yeast Atg1) in diabetic mice (Figure 6(a)). The phosphorylation levels of AMPK and ULK1 were decreased in the diabetic group compared with the control group with or without DHM treatment. DHM treatment enhanced the phosphorylation of AMPK and ULK1 in diabetic mice, which suggests that DHM prevents cardiac dysfunction in diabetic mice by restoring autophagy through AMPK/ULK1 activation (Figures 6(f) and 6(g)).

## 4. Discussion

In this study, we demonstrated that DHM treatment could ameliorate the progression of DCM in streptozotocin-induced diabetic mice. The effects of DHM against DCM were associated with reduced oxidative stress and inflammation, improved mitochondrial function, inhibited cardiac apoptosis, and restored cardiac autophagy in diabetic mice. Moreover, the DHM treatment enhanced the phosphorylation of AMPK and ULK1 of diabetic mice, which suggests that DHM significantly enhanced autophagy to protect against cardiac dysfunction in diabetic mice through AMPK/ULK1 activation. These findings strongly suggest that DHM is a potential therapeutic agent to treat DCM.

ROS is overproduced in both type 1 and type 2 diabetes [14]. Increased ROS production in the diabetic heart is a contributing factor in the development and progression of DCM. Koncsos et al. reported that mild diastolic dysfunction and cardiac hypertrophy are associated with elevated oxidative stress in prediabetes, and prediabetes-induced oxidative stress originates from the subsarcolemmal mitochondria [15]. When ROS generation and ROS-degrading pathways are imbalanced, superoxide-mediated damage and cellular dysfunction will accumulate [16, 17]. Antioxidant defenses could contribute to oxidative stress in the diabetic myocardium [18]. In the present study, decreased level of MDA and increased activities of SOD and GSH-Px were detected in the diabetic group, which is consistent with the literature. Moreover, DHM administration lowered MDA content by upregulating the activities of SOD and GSH-Px. Cardiac inflammation is one of the hallmarks of the development of DCM. Clinical and experimental studies have suggested that enhanced cardiac inflammation in diabetes might exacerbate DCM [19]. We are the first to report that levels of IL-6 and TNF-*α* were suppressed by DHM in diabetic mice.

Structural injuries and functional impairment of the mitochondria contribute to diabetic heart disease. Studies of mitochondria have reignited interest in a role for mitochondrial dysfunction in the pathogenesis of DCM [20]. Kuo et al. first reported impaired mitochondrial function in *db*/*db* heart mitochondria 25 years ago [21]. Mitochondria from the hearts of type 1 diabetic animals show reduced oxidative phosphorylation capacity and exhibit lower creatine phosphate activity, lower ATP synthase activity, and creatine-stimulated respiration [17]. In our study, transmission electron microscopy revealed swollen and disordered mitochondria in the myocardium of diabetic mice. The administration of DHM alleviated mitochondrial ultrastructure impairment, as evidenced by intact membranes and cristae in the diabetic mice. Simultaneously, DHM treatment improved ATP content, CS activity, and complex Ι/ΙΙ/ΙΙΙ/ΙV/V activities in the isolated mitochondria of diabetic mice and enhanced mitochondrial potential in cardiomyocytes subjected to high glucose injury.

Oxidative stress and inflammation-induced apoptosis play critical roles in DCM [22]. Apoptosis helps induce the loss of contractile cells, maximizes cardiac injury, and therefore accelerates the occurrence of heart failure [23]. In the present study, DHM treatment decreased the percentage of TUNEL-positive cardiomyocytes both in the hearts of diabetic mice and in high glucose-treated cells. DHM treatment did not affect the myocardium of normal mice or cardiomyocytes cultured in low glucose. Moreover, the expression of caspase enzymes and the apoptosis-related Bcl-2 family were investigated to demonstrate the molecular basis of the effects of DHM. DHM treatment decreased the levels of cleaved caspase-3 and cleaved caspase-9 in the myocardium of diabetic mice. Regarding the apoptosis-related Bcl-2 family proteins, decreased levels of Bax and increased levels of Bcl-2 resulted in a downregulated Bax/Bcl-2 ratio in diabetic mice with DHM treatment.

Autophagy is a cellular housekeeping process that is essential for removing damaged or unwanted organelles, protein, and lipid aggregates. It is a dynamic process that is tightly regulated by the availability of nutrients and the metabolic balance of the cell, and functional autophagy is indispensable for cellular survival in low-energy conditions [24]. In the heart, constitutive autophagy is not only a homeostatic mechanism for maintaining cardiac structure and function but is also of great importance for the maintenance of cardiac cellular integrity. However, disruption of autophagy may lead to heart failure [25]. Autophagy is inhibited in the hearts of type 1 diabetic mice, which indicates that inhibiting autophagy may be associated with the development of DCM [26]. Moreover, this inhibition may be an adaptive response that limits cardiac dysfunction in type 1 diabetes, presumably through upregulation of alternative autophagy and mitophagy [27, 28]. DHM modulates autophagy in many other circumstances, as mentioned above. DHM improves skeletal muscle insulin resistance by inducing autophagy via the activation of AMPK via a decrease in the cellular AMP/ATP ratio, resulting from F0F1-ATPase inhibition [9]. DHM also inhibits the cell proliferation in HepG2 cells through enhancing autophagy [29]. Consistently, in the present study, evidence for the suppression of autophagic activity in the hearts of STZ-induced diabetic mice was uncovered by increased LC3 ΙΙ/LC3 Ι ratio and Atg7 and Beclin1 protein levels. Cardiomyocytes cultured under high glucose often display less GFP-LC3 puncta and more accumulation of aggresomes and P62, which indicate insufficient autophagy compared with cells cultured under low glucose conditions. Interestingly, the administration of DHM significantly enhanced autophagy, as evidenced by the LC3 ΙΙ/LC3 Ι ratio, the expression of Beclin1 and Atg7 of diabetic mice, the increased GFP-LC3 puncta, and the reduced accumulation of aggresomes and P62 in cardiomyocytes cultured under high glucose conditions.

AMPK is a major regulator of cellular and whole-body energy homeostasis. It is activated in response to an increase in the intracellular AMP-to-ATP ratio during exercise, hypoxia, oxidative stress, and glucose deprivation. Emerging evidence demonstrates that AMPK regulates not only cellular energy but also other cellular processes, such as cell growth, protein synthesis, and autophagy [30]. A recent study reported that the dissociation of Bcl-2 from Beclin1 via activated AMPK enhances cardiac autophagy and protects against cardiomyocytes in diabetes [26]. In our current study, STZ-induced DCM decreased AMPK activity and subsequently suppressed cardiac autophagy, which in line with the finding from OVE26 type 1 diabetic mice [31]. Moreover, AMPK directly regulates autophagy by phosphorylating and activating ULK1 [30]. Our study showed that the decreased AMPK activity in STZ-induced diabetic mice was accompanied by decreased ULK1 phosphorylation. The administration of DHM enhanced AMPK and ULK1 phosphorylation in the hearts of STZ-induced diabetic mice. Together, these data suggest that DHM protects against DCM in STZ-induced diabetic mice by restoring autophagy through AMPK/ULK1 activation.

In summary, our study suggests that DHM may have great therapeutic potential in the treatment of DCM. The effects of DHM against DCM may be associated with its pharmacological activities, including antioxidant and anti-inflammatory activity. DHM also attenuated cardiac apoptosis and normalized cardiac autophagy, which may be beneficial in preventing DCM.

## Figures and Tables

**Table 1:** Changes in general characteristics before and after 14 weeks of treatment with DHM in STZ-induced diabetic mice.

	CON	CON + DHM	DM	DM + DHM
Blood glucose (mmol/L) (0 day)	8.36 ± 1.77	8.13 ± 1.48	22.15 ± 2.18^*∗*#^	22.56 ± 2.25^*∗*#^
Blood glucose (mmol/L) (14 weeks)	8.54 ± 1.26	8.29 ± 0.84	32.27 ± 1.84^*∗*#^	31.26 ± 0.98^*∗*#^
Body weight (g) (14 weeks)	30.25 ± 1.86	31.26 ± 1.69	18.69 ± 2.13^*∗*#^	26.13 ± 1.85^*∗*#$^
Food consumption (g/kg/d) (14 weeks)	135.36 ± 11.20	138.29 ± 9.80	383.35 ± 18.96^*∗*#^	358.23 ± 24.56^*∗*#^
Water intake (ml/kg/d) (14 weeks)	265.39 ± 28.89	278.36 ± 21.42	1209.23 ± 41.32^*∗*#^	1103.84 ± 35.65^*∗*#^

The data are presented as means and SD (*n* = 6). ^*∗*^*P* < 0.05 versus CON; ^#^*P* < 0.05 versus CON + DHM; ^$^*P* < 0.05 versus DM.
